# A dual role for pleiotrophin in modulating inflammation and myelination in the presence of chondroitin sulfate proteoglycans after nervous system injury

**DOI:** 10.3389/fncel.2025.1549433

**Published:** 2025-02-27

**Authors:** Somnath J. Gupta, Matthew A. Churchward, Kathryn G. Todd, Ian R. Winship

**Affiliations:** ^1^Neurochemical Research Unit, Department of Psychiatry, Faculty of Medicine and Dentistry, University of Alberta, Edmonton, AB, Canada; ^2^Neuroscience and Mental Health Institute, University of Alberta, Edmonton, AB, Canada; ^3^Department of Biological Sciences, Concordia University of Edmonton, Edmonton, AB, Canada

**Keywords:** stroke, CSPGs, OPCs, oligodendrocytes, microglia, PTN

## Abstract

Chondroitin sulfate proteoglycans (CSPGs), key components of the extracellular matrix and the glial scar that forms around central nervous system (CNS) injuries, are recognized for hindering neuronal regeneration. We previously demonstrated the potential of pleiotrophin (PTN) to induce neurite outgrowth even in the presence of inhibitory CSPGs. The effects of PTN on microglia and oligodendrocytes are not well described. Here, we examined how PTN administration alters the differentiation of oligodendrocyte precursor cells (OPCs) into mature oligodendrocytes in the presence of CSPGs using *in vitro* cell culture model. Moreover, we explored the effects of PTN on the inflammatory activity of microglia with and without inflammatory stimulation (IFN-*γ*) in a CSPG-rich environment. The data showed that the CSPG matrix inhibited the differentiation of OPCs into mature oligodendrocytes. PTN induced dose-dependent differentiation of OPCs into mature oligodendrocytes, with an optimal effect at 10 nM PTN. Moreover, PTN modified the immunological response of microglia in the presence of CSPGs, with reduced proinflammatory activity that was further reduced by PTN administration, in contrast to the increased release of matrix metalloproteinases (MMP 9). However, when IFN-*γ*-activated microglia were treated with PTN, proinflammatory signaling was stimulated at higher PTN concentrations (10 nM and 100 nM). Overall, our results indicate that PTN can overcome the inhibitory effect of CSPGs on the differentiation of OPCs into oligodendrocytes and can modulate inflammation mediated by CSPGs from microglia. Collectively, these findings demonstrate that PTN can effectively counteract the inhibitory effects of CSPGs on the differentiation of OPCs into mature oligodendrocytes while also modulating microglial responses to reduce proinflammatory activity and increase MMP-9 release. Thus, PTN has great potential to improve remyelination and neuroprotective strategies in the treatment of demyelinating diseases or any injury.

## 1 Introduction

Neurons, the fundamental building blocks of the central nervous system (CNS), are specialized cells responsible for transmitting information throughout the body via electrical and chemical signaling pathways (Fünfschilling et al., 2012; Kuboyama et al., 2017). This transmission of information is impaired by CNS injury due to initial damage and programs of gene expression after injury, which can limit the ability of the CNS to regenerate. Key processes that limit recovery include the buildup of toxic compounds that drive continuous damage in or near sites of injury, increased expression of factors that inhibit growth, the formation of a glial scar around injured tissue, and the modulation of the endogenous immune response from microglia (Bergles and Richardson, 2016; El Waly et al., 2014; Lee et al., 2012).

Chondroitin sulfate proteoglycans (CSPGs) are key components of the extracellular matrix that act as key guidance cues during neurodevelopment and preserve CNS stability in the uninjured brain (Siebert et al., 2014; Morawski et al., 2004). However, after injury, CSPG production is upregulated in several CNS cell types and leads to the generation of a CSPG-rich glial scar and upregulation in the adjacent extracellular matrix (Tran et al., 2022; Fawcett and Asher, 1999; Pu et al., 2018). CSPGs in the glial scar act as a barrier to restrict the spread of injury (Siebert et al., 2014; Rauch and Kappler, 2006; Djerbal et al., 2017; Sami et al., 2020), but increased CSPGs in the scar and adjacent tissue also inhibit the axonal regeneration and remyelination processes that are important for restoring neurotransmission (Pendleton et al., 2013; Sun et al., 2017).

CNS injury is associated with demyelination of axons around the injury site (Huntemer-Silveira et al., 2021; Coutinho Costa et al., 2023). Thus, remyelination after CNS injury is essential for restoring high-fidelity neurotransmission and is dependent on the migration and differentiation of oligodendrocyte precursor cells (OPCs) (Leenders et al., 2024; Marangon et al., 2024; Ma et al., 2024). After injury, OPCs migrate to the damaged area and may differentiate into mature oligodendrocytes that can remyelinate neurons (Raffaele and Fumagalli, 2022; Kim et al., 2023). However, this remyelination process is inefficient and fails to effectively myelinate all the developing or regenerating neurons, as the OPCs become stalled during differentiation (de Faria et al., 2019). Several factors affect the differentiation of OPCs, including insufficient access to growth and differentiation factors, increased inflammation, and changes to the extracellular matrix from glial scar components, including CSPGs (Bergles and Richardson, 2016; El Waly et al., 2014; Lee et al., 2012).

Inflammation plays a major role in determining the fate of the differentiation of OPCs into mature oligodendrocytes. Activated microglia release CSPGs as well as proinflammatory cytokines (Fawcett and Asher, 1999; Raffaele and Fumagalli, 2022). During the early stage of injury, microglia migrate to the injury site and release proinflammatory cytokines to activate astrocytes to form a glial scar (Raffaele and Fumagalli, 2022). Notably, early after injury, microglia-mediated tumor necrosis factor (TNF) release potentiates the phagocytic signaling necessary for the clearance of myelin debris and the generation of new oligodendrocytes (Cunha et al., 2020; Hammel et al., 2022; Kotter et al., 2006). However, *in vitro* data suggest reduced release of the proinflammatory cytokines TNF and nitric oxide (NO) from microglia in response to treatment with LPS when microglia are in the presence of CSPGs, suggesting that CSPGs reduce the proinflammatory activity of microglia (Rolls et al., 2008). Thus, during the early stages of injury, the inflammatory activity of microglia potentiates the removal of myelin debris and the generation of oligodendrocytes. However, increased accumulation of CSPGs in later stages of recovery might reduce the release of microglial cytokines that support remyelination and directly inhibit the differentiation of OPCs. Thus, inflammation and CSPGs play dual roles during the acute or later stages of CNS injury.

Strategies to enhance OPC differentiation while preserving the protective effects of CSPGs could involve neutralizing CSPG inhibitory activity without disrupting CSPG synthesis. One potential agent that can modify CSPG signaling while enhancing the differentiation of oligodendrocytes is pleiotrophin (PTN). PTN is involved in the early developmental phase of neuritogenesis, synaptogenesis, and memory formation (Tang et al., 2019). PTN expression is reduced in later phases of development, but the expression increases during an injury (González-Castillo et al., 2015; Nikolakopoulou et al., 2019; Mi et al., 2007) and is postulated to be involved in restoring neuronal outgrowth overcoming the inhibition mediated by CSPGs (Gupta et al., 2023). PTN promotes the differentiation and proliferation of OPC-like OL-1 cells by inactivating the CSPG receptor PTPRZ (McClain et al., 2012). PTN also impacts the activity of microglia, causing the release of several neurotrophic factors (Miao et al., 2012). Notably, PTN promotes the release of proinflammatory cytokines (TNF, IL-6 and MCP-1) from LPS-stimulated microglia (Fernández-Calle et al., 2017). Thus, PTN induces a proinflammatory effect in activated microglia, whereas CSPGs suppress the proinflammatory effect on LPS-stimulated microglia (Fernández-Calle et al., 2017; Rolls et al., 2008). Thus, stimulating microglia in a CSPG-rich environment reduced the release of proinflammatory cytokines, whereas PTN treatment of microglia potentiated the release of proinflammatory cytokines in an environment devoid of CSPGs. Under *in vivo* conditions, cells in the glial scar are always under the influence of CSPGs, yet no studies have focused on identifying the effect of the PTN on microglia in CSPG-rich environments. Moreover, while previous studies have identified the effects of CSPGs or PTN in microglia stimulated with LPS, a more relevant simulation protocol for CNS injury involves creating a proinflammatory environment via treatment with IFN*γ*.

Here, we directly investigated the potential role of PTN in modulating the differentiation of OPCs to oligodendrocytes *in vitro* and the inflammatory activity of microglia in the presence of CSPGs. Our data revealed a dose-dependent effect of PTN on the differentiation of OPCs into oligodendrocytes, suggesting that PTN might be a strategy to increase remyelination. Moreover, in the presence of CSPGs, PTN can enhance the proinflammatory stimulus from activated microglia (IFN γ-stimulated microglia) in an environment mimicking early CNS injury. However, PTN reduces proinflammatory activity in unstimulated microglia, and this reduction in inflammation later in the recovery process could support recovery and remyelination.

## 2 Materials and methods

### 2.1 Matrix preparation

For the OPC study, 12 mm coverslips (Fisherbrand, 12–545-81) were coated with 100 μg mL^−1^ poly-D-lysine (Sigma–Aldrich, P6407) for 2 h at 37°C and 5% CO_2_. After three washes with water, the coverslips were coated with growth inhibitory matrix containing 1.0 μg/mL laminin +5 μg/mL CSPGs (Sigma Aldrich, CC117) for 2 h and then washed with PBS. For the microglial study, coverslips were coated with a growth permissive matrix of 100 μg mL^−1^ poly-L-lysine (Sigma–Aldrich, P6282) for 2 h or an inhibitory matrix with 5 μg mL^−1^ CSPGs (Sigma–Aldrich, CC117) at 37°C and 5% CO_2_ (for OPC study). After three washes with water, the coverslips were coated with 1.0, 2.5, or 5 μg/mL CSPGs for 2 h and washed 2 times with PBS.

### 2.2 Primary OPC cell culture

All animal experiments were conducted in accordance with the Canadian Council on Animal Care Guidelines and approved by the Animal Care and Use Committee: Health Sciences for the University of Alberta. Mouse primary OPC cell cultures were isolated from 2-day-old heterozygous CX3CR1-eGFP mice. The cortices were dissected and digested with TrypLE (Gibco, 12,605--028) for 10 min at 37°C. The tissues were treated with 60 μg mL^−1^ DNaseI (Sigma, DN25-100) for 5 min and centrifuged at 500 × g for 3 min. The cells were dissociated from the tissue by trituration in Dulbecco’s modified Eagle’s medium nutrient mixture/Ham’s F-12 (DMEM/F12) (Gibco, 11,320–033) supplemented with 10% fetal bovine serum (Gibco, 12,483–020) and 1% penicillin/streptomycin (Gibco, 15,140–122). The cells were then centrifuged and cultured in T25 flasks coated with 100 μg mL^−1^ poly-L-lysine (Sigma–Aldrich, P6282) at 37°C and 5% CO_2_. 2/3rd media was changed every 3 days; on day 9, the flasks were placed on a shaker at 50 rpm at 37°C and 5% CO_2_ to remove any loosely adherent cells. The media was changed, and the flasks were placed on a shaker at 220 rpm for 16 h at 37°C and 5% CO_2_ to detach the OPCs. The media were collected, and the media with OPCs were then incubated in a cell culture dish for 30 min with gentle agitation every 15 min. All the floating cells were OPCs, and the adherent cells were microglia. OPCs were isolated by centrifugation at 1200 rpm for 10 min and seeded at 3 × 10^4^ cells/well onto coverslips coated with different matrices in OPC proliferation medium [DMEM/F12 (Gibco, 11,320–033), B27 supplement (1:50 v/v) (Gibco, 17,504–044), 20 ng/mL platelet-derived growth factor (PDGF-AA, Peprotech, 100–13A–10UG), 20 ng/mL fibroblast growth factor basic (FGF basic Peprotech, 100–18B–10UG), or 5 μg/mL insulin (I6634, Sigma–Aldrich)] for 24 h. After incubation, the medium was changed to OPC differentiation media [DMEM/F12 (Gibco, 11,320–033), B27 supplement (1:50 v/v) (Gibco, 17,504–044), N-acetyl-L-cysteine (NAC; Sigma–Aldrich, A9165-5G), ciliary neurotrophic factor (CNTF; Peprotech, 450–13-20UG), 30 ng mL^−1^ thyronine (3,3′,5-triiodo-L-thyronine sodium salt; Sigma–Aldrich, T2752), 30 ng mL^−1^ thyroxine (L-thyroxine sodium salt pentahydrate Sigma–Aldrich, T0397) and different concentrations (1 nM, 10 nM and 100 nM PTN) of recombinant mouse PTN (R&D 6580-PL-050)]. The PTN concentration used in this study is 1 nM, 10 nM and 100 nM PTN corresponding to 0.018, 0.18, 1.8 μg/mL PTN which is lower than those previously used *in vivo* concentration of 1.5 μg PTN (Paveliev et al., 2016), so we are not depending on excessively high doses to produce this effect, which supports its translational potential. The cells were incubated for 7 days, with ½ media changed every day. The cells were then fixed with 5% formaldehyde for 20 min, washed with PBS and processed for immunocytochemistry.

### 2.3 Primary microglial cell culture

Primary microglia were isolated from postnatal day 2 heterozygous CX3CR1-eGFP mice. The cortices were dissected and digested with TrypLE (Gibco, 12,605--028) for 10 min at 37°C. The tissues were treated with 60 μg mL^−1^ DNaseI (Sigma, DN25-100) for 5 min and centrifuged at 300 × g for 5 min. The cells were dissociated from the tissue by trituration in Dulbecco’s modified Eagle’s medium Ham’s nutrient mixture F-12 (DMEM/F12) (Gibco, 11,320–033) supplemented with 10% fetal bovine serum (Gibco, 12,483–020) and 1% penicillin/streptomycin (PS) (Gibco, 15,140–122). The cells were subsequently centrifuged at 500 × g for 3 min and cultured in 12-well cell culture plates coated with 100 μg mL^−1^ poly-L-lysine (PLL) (Sigma–Aldrich, P6282) at 37°C and 5% CO_2_. The cells were cultured for 19 days at 37°C and 5% CO_2_, and the media was changed twice weekly. On the 20^th^ day, for microglial isolation, confluent cell layers were washed with sterile PBS and treated with a mixture of trypsin–EDTA (0.25%) (Gibco, 25,200,072) and DMEM F12 (1:3) for 20 min. The isolated cell layer consisted of a mixed cell population, and the cells attached to the plates were pure microglia. Microglia were trypsinized with 0.25% trypsin–EDTA for 10 min, centrifuged at 1200 rpm for 10 min and seeded at 1 × 10^5^ cells/well onto coverslips coated with different matrices for 24 h in DMEM F12/1%PS. After 24 h, the media was changed to media containing different concentrations of recombinant mouse PTN (R&D 6580-PL-050) for 72 h. The media was collected after 72 h and analyzed for cytokine levels via a proinflammatory focused 10 plex discovery assay (Eve Technologies, Calgary, AB), and the cells were fixed after 72 h of treatment with 5% formaldehyde solution for 15 min, washed with PBS and processed for immunocytochemistry. For cytokines analysis, the released cytokines were normalized to the total amount of protein present in each sample and then corrected with PLL control with the levels released in PLL control as 100%.

### 2.4 EdU proliferation assay

Microglial proliferation was measured via EdU analysis via a Click-iT EdU kit (Thermo Fisher Scientific, C10638). Briefly, microglia were added onto coverslips coated with poly-L-lysine as a control and with different concentrations of CSPGs (1, 2.5 and 10 μg mL^−1^). After 24 h of incubation, the media was changed to media containing 20 μM EdU and different concentrations of PTN with or without 100 μg/mL IFN. After 72 h, the cells were fixed with 5% formaldehyde for 15 min and washed 3 times with PBS. Following fixation, the cells were permeabilized with 0.5% Triton X-100. The Click-iT mixture was added to each well and incubated for 30 min. Following incubation, the cells were washed twice with 0.1% Triton X-100 in PBS. The cells were then processed for Iba1 immunostaining.

### 2.5 Gel zymography

The conditioned media from the cell cultures were centrifuged to remove cell debris and concentrated via an Amicon Ultra2 centrifugal filter unit (Millipore UFC201024). MMP activity was determined by gel zymography via sodium dodecyl sulfate–polyacrylamide gel electrophoresis (SDS–PAGE) with modifications. The samples were run on a 7.5% acrylamide gel with 0.1% (w/v) gelatin. A total of 15 μg of protein was loaded into each well, diluted 5X sample buffer to 1X buffer and run under nonreducing conditions at 110 V and 4°C for 2 h. After electrophoresis, the gels were washed twice with wash buffer for 30 min with agitation (2.5% Triton X-100, 50 mM Tris HCl, 5 mM CaCl_2_, and 1 μM ZnCl_2_). The gels were then incubated in incubation buffer (1.0% Triton X-100, 50 mM Tris HCl, 5 mM CaCl_2_, 1 μM ZnCl_2_) for 24 h at 37°C. The gels were then stained with 0.5% Coomassie blue (0.5% Coomassie blue, 50% water, 10% acetic acid, 40% methanol) for 1 h. The gels were then destained with destaining solution (40% methanol, 10% acetic acid, 50% water) until white bands were detected under a blue background. The gels were then imaged via a LICOR Odyssey reader. The bands were analyzed via a gel analyzer plugin in Fiji (Schindelin et al., 2012).

### 2.6 Immunocytochemistry

The fixed samples were blocked with 10% normal horse serum in PBS containing 0.1% Triton-X 100 and washed with PBS. The samples were stained overnight with myelin basic protein antibody (MBP) (1:500, Merck Millipore MAB386), oligodendrocyte marker 4 (O4) (1:500, R&D MAB1326), platelet-derived growth factor *α* (PDGFRα) (1:500, R&D AF1062), chondroitin sulfate proteoglycans (CSPGs) CS56 (1:500, Sigma–Aldrich C8035) and Iba1 (1:500, Thermo Fisher Scientific PA5--27436) (PBS pH 7.4 with 0.1% HS, overnight at 4°C). The primary antibodies against CS56 and O4 were detected with the secondary antibody donkey anti-mouse Alexa 647 (1:500 Abcam ab150107), and MBP was detected with donkey anti-rat Alexa 488 (1:500 Life Technologies A21208). PDGFRα was detected with donkey anti-goat Alexa 546 (1:500 Life Technologies, A11056), and Iba1 was detected with the secondary antibody goat anti-rabbit Alexa 488 (1:500 Thermo Fisher Scientific A-11034). The nuclei were stained with Hoechst 33342 (1:1000, Invitrogen, 62,249). The samples were then mounted on slides with Fluormount-G (Southern Biotech). The images were taken via a Leica TCS-SPE fluorescence microscope.

### 2.7 Statistics

Statistical analyses of data from fluorescence images were carried out via one-way ANOVA followed by Tukey’s multiple comparisons test for significance between treatment groups. For SHOLL analysis, the data were analyzed via two-way ANOVA and Tukey’s multiple comparisons test for significance between treatment groups. *n* represents a single independent experiment (i.e., an independent culture preparation) with a minimum of three technical replicates. Each technical replicate represents a well in a 24-well culture plate. All image analyses were performed by experimenters who were blinded to the experimental conditions, and the statistical analyses were performed via GraphPad Prism version 9.1.2.

## 3 Results

### 3.1 PTN drives the differentiation of OPCs into oligodendrocytes, overcoming the inhibitory effect of CSPGs

**Figure 1:**
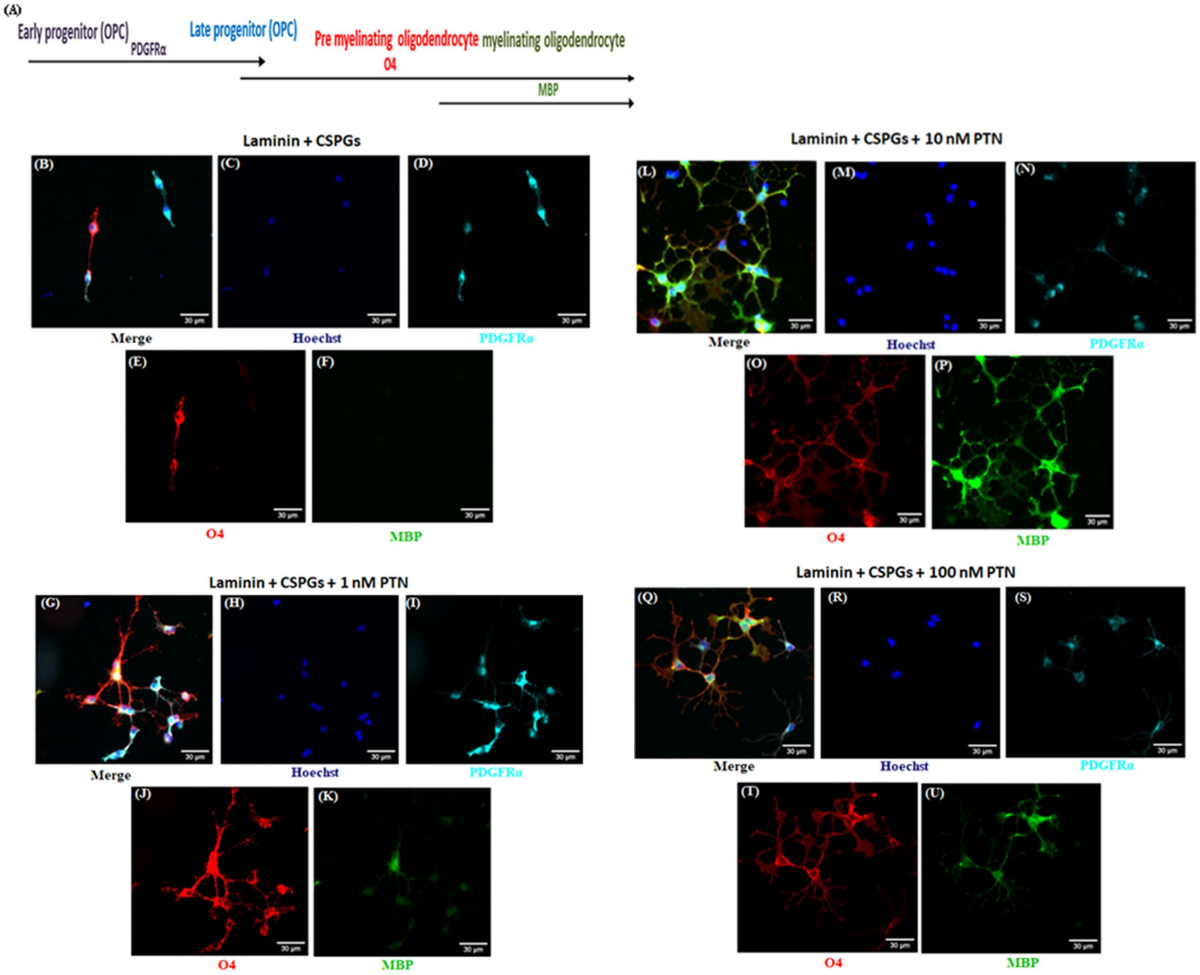
PTN induces the expression of various oligodendrocyte lineage differentiation markers. Isolated OPCs were seeded on laminin and laminin + CSPGs and cultured for 7 days in differentiation medium supplemented with different concentrations of PTN. Fixed cells were stained with anti-PDGFR*α*, anti-O4 and anti-MBP in conjunction with nuclear stain with Hoechst. **(A)** Markers of OPCs and oligodendrocytes during the differentiation process. Epifluorescence images of differentiating OPC cultures were acquired at 20X. OPCs were identified with the marker PDGFRα (cyan), premyelinating oligodendrocytes with O4 (red), and differentiating oligodendrocytes with MBP (green). **(B–F)** OPCs cultured on the laminin + CSPG matrix, **(G–K)** laminin + CSPG matrix and treated with 1 nM PTN, **(L–P)** laminin + CSPG matrix and treated with 10 nM PTN, and **(Q–U)** laminin + CSPG matrix and treated with 100 nM PTN. The scale bar represents 30 μm.

To determine the effect of PTN on the differentiation of OPCs in the presence of CSPGs, we coated coverslips with laminin and CSPGs. The isolated OPCs were seeded onto the matrix and cultured in the presence or absence of different concentrations of PTN (1 nM, 10 nM, or 100 nM) for 7 days. To measure the differentiation of OPCs into oligodendrocytes, the cells were fixed after 7 days of treatment and immunolabeled with markers of the oligodendrocyte lineage (OPCs, premyelinating oligodendrocytes and differentiated/mature oligodendrocytes). During the differentiation of OPCs into oligodendrocytes, protein expression gradually changes, with OPCs expressing PDGFRα, premyelinating oligodendrocytes expressing O4 and mature oligodendrocytes expressing MBP (Figure 1A). OPCs differentiate and express PDGFRα and O4 even in the presence of CSPGs, but do not differentiate into oligodendrocytes expressing MBP (Figures 1B–K). PTN enhanced the differentiation of OPCs into oligodendrocytes in a dose-dependent manner. OPCs differentiated into oligodendrocytes, as indicated by enhanced expression of O4 and MBP, upon treatment with 1 nM PTN (Figures 1G–K), 10 nM PTN (Figures 1L–P) or 100 nM PTN (Figures 1Q–U).

**Figure 2:**
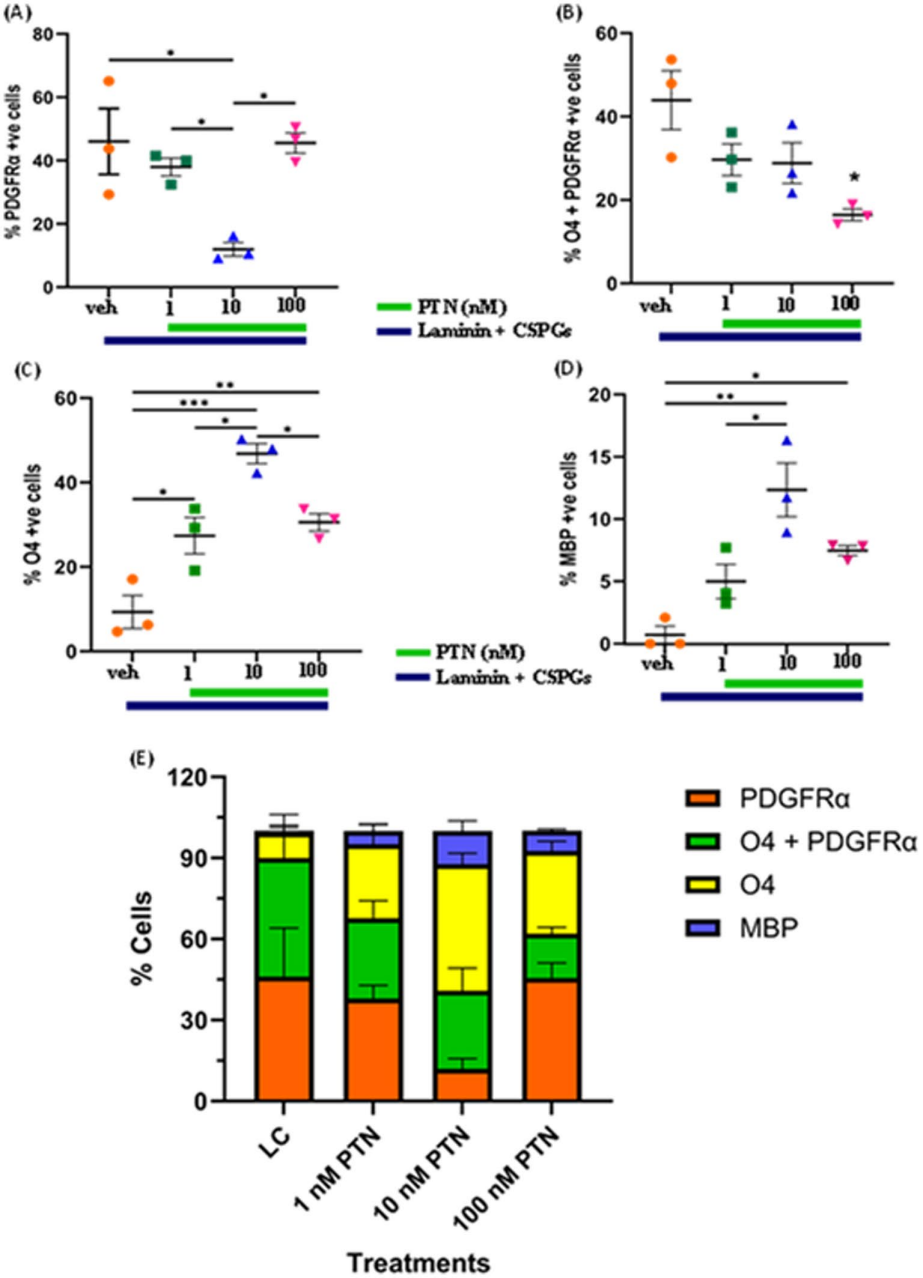
Effect of PTN on the differentiation of OPCs into oligodendrocytes. OPCs were cultured on laminin + CSPG (LC) matrix and treated with different concentrations of PTN for 7 days. The cells were stained for PDGFR α, O4 and MBP, and those positive for PDGFR α, O4 + PDGFR α, O4 and MBP were counted and plotted as percentages of the total cell population. **(A)** PTN induces the differentiation of OPCs by reducing the number of PDGFR α cells (ANOVA, F(3, 8) = 7.831 *p* = 0.0091), favoring the generation of **(B)** O4 + PDGFR α-expressing cells (ANOVA, F_(3, 8)_ = 5.635 *p* = 0.0226), **(C)** O4-expressing cells (ANOVA, F_(3, 8)_ = 21.59 *p* = 0.0003), **(D)** MBP-expressing cells (ANOVA, F_(3, 8)_ = 13.17 *p* = 0.0018). Oligodendrocyte lineage marker-positive cells expressing PDGFR α, O4 + PDGFR α, O4 and MBP in **(E)** Cell population distribution across different lineages of the OPC differentiation process with 1 nM, 10 nM and 100 nM PTN treatment. **(A–D)** Error bars represent the standard error of the mean (SEM). After ANOVA, Tukey’s *post hoc* test was performed on the datasets. The symbols *, ** and ***/**** represent *p <* 0.05, 0.01, and 0.001, respectively. All the data are based on 3 independent experiments with a minimum of three technical replicates.

The quantification of lineage markers revealed a dose-dependent effect of PTN treatment on PDGFRα-positive cells (ANOVA, F_(3, 8)_ = 7.831 *p* = 0.0091) (Figure 2A). *Post hoc* comparisons revealed that, compared with vehicle treatment, 10 nM PTN treatment significantly reduced the number of PDGFRα-positive cells compared to vehicle (Tukey’s *p =* 0.0127), 1 nM PTN treatment (Tukey’s *p =* 0.0496), and 100 nM PTN treatment (Tukey’s *p =* 0.0138). PTN treatment reduced the number of cells expressing both PDGFRα + O4-positive cells (F_(3, 8)_ = 5.635 *p* = 0.0226) (Figure 2B), with *post hoc* comparisons identified a peak response for 100 nM PTN treatment significantly reduced the number of PDGFRα- and O4-positive cells compared with that in the nontreated condition (Tukey’s *p* = 0.0145). Notably, the premyelinating oligodendrocyte population expressing O4 significantly increased with PTN treatment (F_(3, 8)_ = 21.59 *p =* 0.0003) (Figure 2C), and post hoc analysis revealed that compared with the vehicle control, 10 nM PTN significantly increased the O4-positive cell population (Tukey’s *p =* 0.0002), 1 nM PTN (Tukey’s *p =* 0.0135) and 100 nM PTN (Tukey’s *p =* 0.0339). MBP-expressing mature oligodendrocytes significantly increased with PTN treatment (F_(3, 8)_ = 13.17 *p =* 0.0018) (Figure 2D), and post hoc analysis revealed that 10 nM PTN significantly increased the MBP-positive cell population compared with the vehicle control (Tukey’s *p =* 0.0012) and 1 nM PTN (Tukey’s *p =* 0.00195).

Figure 2E summarizes the distribution of cell lineage markers under different treatment conditions. In the absence of PTN, there was no significant difference in the percentages of cells expressing PDGFRα (46.04% of the total cell population) and those positive for PDGFRα and O4 (43.93% of the total cell population), but there were significantly fewer premyelinating O4-expressing cells (9.32% of the total cell population) and MBP-expressing cells (0.69% of the total cell population). Treatment with 1 nM PTN did not significantly increase the percentage of cells expressing PDGFRα (37.97% of the total cell population) or O4-expressing premyelinating cells (27.39% of the total cell population) with fewer MBP expressing cells (4.97% of the total cell population). 10 nM PTN treatment, significantly increased cell population expressing O4 (46.84% of the total cell population) compared to cell population expressing PDGFRα (11.98% of the total cell population) also increased cells expressing MBP (12.33% of the total cell population). 100 nM PTN treatment, significantly increased cell population expressing PDGFRα O4 (45.54% of the total cell population) compared to cell population of cells expressing O4 (16.45% of the total cell population) also increased the number of cells expressing MBP (7.46% of the total cell population). The above data suggest that PTN drives the differentiation of OPCs into oligodendrocytes, overcoming the inhibitory effect of CSPGs.

### 3.2 Effect of PTN on process outgrowth from OPCs and oligodendrocytes

**Figure 3:**
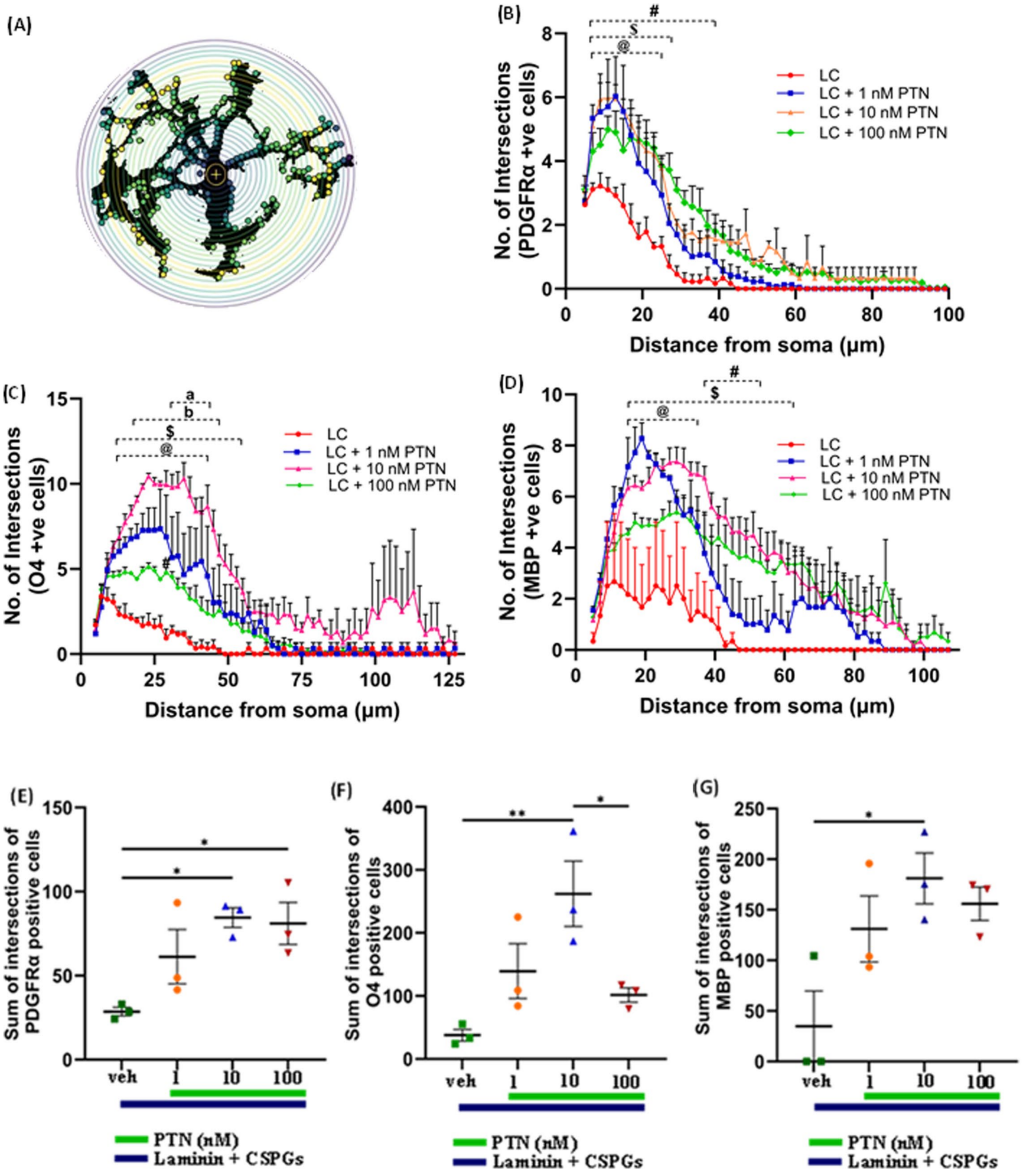
PTN promotes cellular process outgrowth from oligodendrocytes and OPCs. **(A)** Schematic representation of an 8-bit image of a cell with concentric circles around cell bodies via SHOLL analysis. The yellow dots represent the intersections at that radius. **(B)** PTN treatment significantly increased the number of intersections for PDGFR α-positive cells. Two-way ANOVA revealed a significant interaction effect between the number of intersections and treatment for PDGFR α-positive cells (F_(141, 384)_ = 1.757 *p <* 0.0001), a significant main effect on the number of intersections (F_(47, 384)_ = 59.84 *p <* 0.0001) from the soma and treatment (F_(3, 384)_ = 79.15 *p <* 0.0001) for PDGFR α-positive cells. Tukey’s post hoc test revealed a significant difference between different concentrations of PTN. **(C)** PTN treatment significantly increased the number of intersections for O4-positive cells. Two-way ANOVA revealed a significant interaction effect between the number of intersections and treatment for O4-positive cells (F_(183, 496)_ = 1.9 *p <* 0.0001), a significant main effect on the number of intersections (F_(61, 496)_ = 19.29 *p <* 0.0001) from the soma and treatment (F_(3, 496)_ = 163.1 *p <* 0.0001) for O4-positive cells. Tukey’s post hoc test revealed a significant difference between the different concentrations of PTN. **(D)** PTN treatment significantly increased the number of intersections for MBP-positive cells. Two-way ANOVA revealed a significant interaction effect between the number of intersections and treatment for MBP-positive cells (F_(153, 416)_ = 1.290 *p =* 0.0249), a significant main effect on the number of intersections (F_(51, 416)_ = 15.14 *p <* 0.0001) from the soma and treatment (F_(3, 416)_ = 94.29 *p <* 0.0001) for MBP-positive cells. Tukey’s post hoc test revealed a significant difference between different concentrations of PTN. PTN treatment significantly increased the number of intersections for **(E)** PDGFR α-positive cells (F_(3, 8)_ = 5.732 *p =* 0.0216), **(F)** O4-positive cells (F_(3, 8)_ = 7.444 *p =* 0.0106) and **(G)** MBP-positive cells (F_(3, 8)_ = 5.147 *p =* 0.0284). **(B–D)** symbols represent @—compared with LC + 1 nM PTN vs. the LC control, $ represents—compared with the LC + 10 nM PTN vs. LC control, and # represents—compared with LC + 100 nM PTN vs. LC control **(B–G)**. The error bars represent the standard error of the mean (SEM), and the symbols *, **, and ***/**** represent *p* < 0.05, 0.01, and 0.001, respectively. Additionally, a, b, @, $, and # represent *p <* 0.05. All the data are based on 3 independent experiments with a minimum of three technical replicates.

To determine the ability of PTN to increase the complexity of outgrowth from OPCs, premyelinating oligodendrocytes and oligodendrocytes, OPCs were isolated from mice pups, cultured on laminin + CSPGs matrix, and treated with different concentrations of PTN (1 nM PTN, 10 nM PTN, or 100 nM PTN). The cells were immunostained for PDGFRα, O4 and MBP, and images were then analyzed for cellular outgrowth using Sholl analysis from Fiji (Ferreira et al., 2014). Intersections were counted every 2 μm, as shown in Figure 3A. PTN treatment significantly increased outgrowth from PDGFRα-positive cells (Figure 3B), O4-positive cells (Figure 3C) and MBP-positive cells (Figure 3D). Two-way ANOVA showed a significant interactions between distance from the soma and treatment for PDGFRα-positive cells (F_(141, 384)_ = 1.757 *p <* 0.0001), O4-positive cells (F_(183, 496)_ = 1.9 *p <* 0.0001), and MBP-positive cells (F_(153, 416)_ = 1.290 *p =* 0.0249). Significant effects on the intersection distance from the soma were detected for PDGFRα-positive cells (F_(47, 384)_ = 59.84 *p <* 0.0001), O4-positive cells (F_(61, 496)_ = 19.29 *p <* 0.0001) and MBP-positive cells (F_(51, 416)_ = 15.14 *p <* 0.0001). Treatment had a significant effect on the number of intersections for PDGFRα-positive cells (F_(3, 384)_ = 79.15 *p <* 0.0001), O4-positive cells (F_(3, 496)_ = 163.1 *p <* 0.0001) and MBP-positive cells (F_(3, 416)_ = 94.29 *p <* 0.0001). Tukey’s *post hoc* test revealed that with PTN treatment, there was a significant change in the number of intersections for PDGFRα-positive, O4-positive and MBP-positive cells within the abovementioned distance from the soma (Figures 3B–D; Table 1). *Post hoc* analysis also showed that for O4-positive cells, there was a significant change in the number of intersections between 1 nM PTN and 10 nM PTN treatments within 17 μm – 45 μm, between 10 nM PTN and 100 nM PTN treatments within 31 μm – 45 μm (Figure 3C). To confirm these findings, we further measured the total number of intersections for each treatment condition for PDGFR*α*-positive cells (Figure 3E), O4-positive cells (Figure 3F), and MBP-positive cells (Figure 3G). PTN treatment significantly increased the number of intersections for PDGFRα-positive cells (F_(3, 8)_ = 5.732 *p =* 0.0216), O4-positive cells (F_(3, 8)_ = 7.444 *p =* 0.0106) and MBP-positive cells (F_(3, 8)_ = 5.147 *p =* 0.0284), and *post hoc* analysis revealed that 10 nM PTN treatment significantly increased the number of intersections for PDGFRα (*p* = 0.0253), O4 (*p =* 0.0078) and MBP (*p =* 0.0261) compared to CSPGs control. Thus, these data indicate that 10 nM PTN induces the growth of OPCs and induces their differentiation into oligodendrocytes.

**Table 1:** Radii of process outgrowth from PDGFR α-, O4- and MBP-positive cells showing significant differences in the number of intersections with PTN treatment.

	PDGFR α	O4	MBP
PTN conc. (nM)	Distance form soma (μm)	Distance form soma (μm)	Distance form soma (μm)
1	7–25	13–43	15–35
10	7–29	13–55	15–65
100	7–39	13	41–53

### 3.3 Effect of PTN on the proliferation and release of proinflammatory cytokines from microglia in the presence of CSPGs

**Figure 4:**
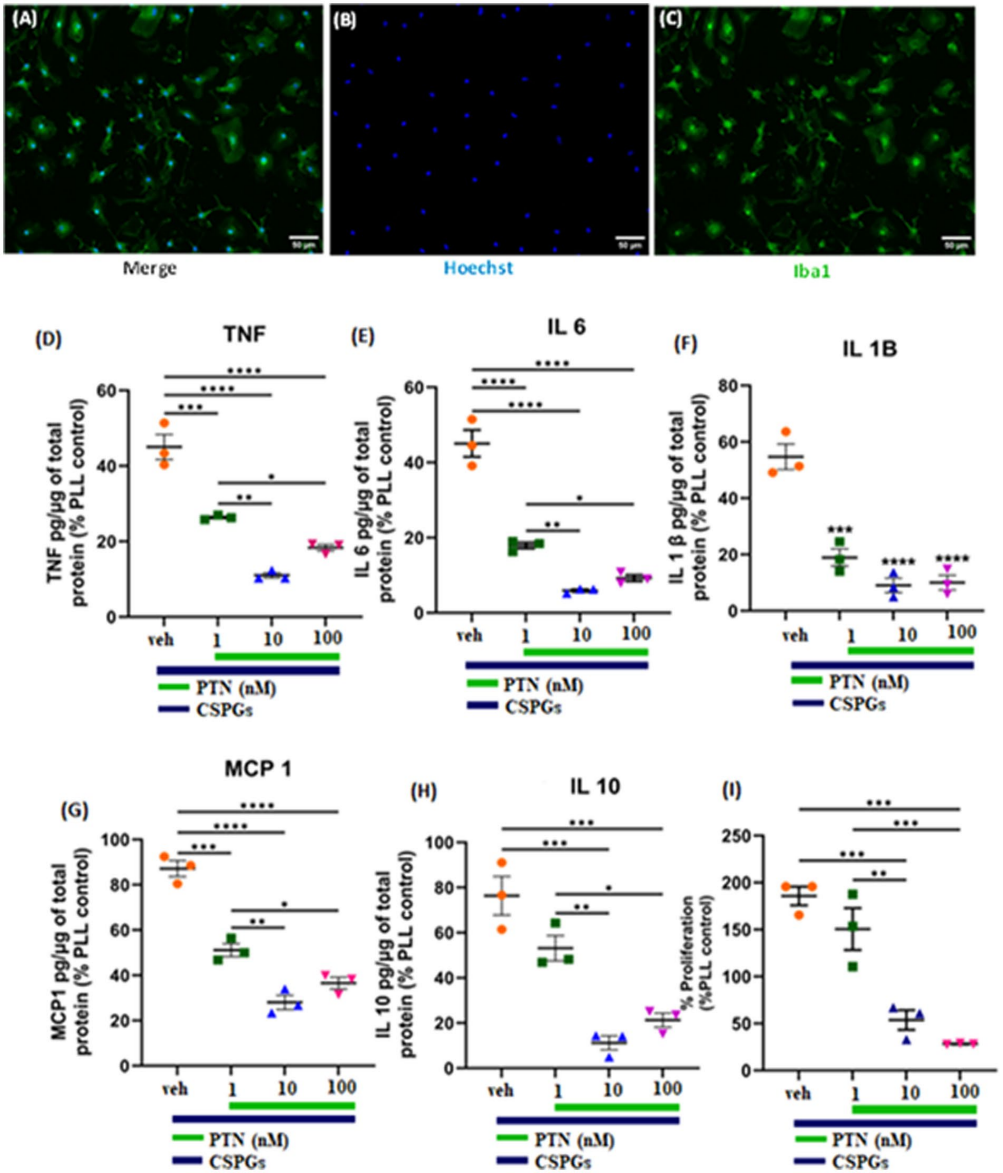
PTN treatment reduced the release of proinflammatory cytokines and the proliferation of microglia. **(A–C)** Microglia were stained with Hoechst and Iba 1. PTN significantly reduced the expression of **(D)** TNF (F_(3, 8)_ = 70.96 *p <* 0.0001), **(E)** IL-6 (F_(3, 8)_ = 87.51 *p <* 0.0001), **(F)** IL-1β (F_(3, 8)_ = 42.89 *p <* 0.0001), **(G)** MCP-1 (monocyte chemoattractant protein 1) (F_(3, 8)_ = 73.51 *p <* 0.0001), **(H)** IL-10 (F_(3, 8)_ = 87.51 *p <* 0.0001), and **(I)** proliferation of microglia (F_(3, 8)_ = 32.18 *p* < 0.0001). **(D–I)** The cytokines released from (poly L Lysine) control were considered 100%, error bars represent the standard error of the mean (SEM), and symbols *, ** and ***/**** represent *p* < 0.05, 0.01, and 0.001, respectively. All the data are based on 3 independent experiments with a minimum of 3 technical repeats.

The cytokines released from microglia can also influence the proliferation and differentiation of OPCs into oligodendrocytes. During an injury, microglia become activated and release cytokines. In this study, the release of cytokines was measured to determine the effect of PTN on microglia in the presence of CSPGs. Microglia isolated from mixed glial cell cultures were first tested for their purity, and, on the basis of immunostaining for Iba1, the isolated cell population was 95% microglia (Figures 4A–C). The microglia were then cultured on a CSPGs matrix (5 μg mL^−1^) and treated with different concentrations of PTN (1 nM, 10 nM, or 100 nM PTN) for 72 h. The media was collected and processed to detect the following cytokines (TNF, IL-6, IL-1B, MCP and IL-10). These data indicate that CSPGs reduce the release of pro-inflammatory cytokines and that, with PTN treatment, the expression of pro-inflammatory cytokines was further reduced. Specifically, in the presence of CSPGs, PTN treatment significantly reduced the expression of TNF-α (F_(3, 8)_ = 70.96 *p <* 0.0001), and *post hoc* analysis further revealed the greatest reduction in the expression of TNF-α at 10 nM PTN (Figure 4D). Similarly, the release of IL-6 was significantly reduced with PTN treatment in the presence of CSPGs (F_(3, 8)_ = 87.51 *p <* 0.0001), with the greatest reduction occurring with the 10 nM and 100 nM PTN treatments (Figure 4E). PTN treatment also reduced the expression of IL-1β in the presence of CSPGs (F_(3, 8)_ = 42.89 *p <* 0.0001) at all different doses of PTN (Figure 4F). PTN treatment reduced the expression of MCP-1 in the presence of CSPGs (F_(3, 8)_ = 73.51 *p <* 0.0001), with peak effects at 10 nM PTN and 100 nM PTN (Figure 4G). A similar pattern of reduced IL-10 expression was detected after PTN treatment (F_(3, 8)_ = 87.51 *p <* 0.0001) (Figure 4H).

In the presence of CSPGs, there was also a significant effect of treatment on the proliferation of microglia (F_(3, 8)_ = 32.18 *p <* 0.0001), with post hoc analysis revealing increased proliferation at 1 nM PTN relative to 10 nM (Tukey’s *p =* 0.0039) and 100 nM PTN (Tukey’s *p =* 0.0009) (Figure 4I).

### 3.4 Effect of PTN on the proliferation and release of proinflammatory cytokines from microglia in the presence of CSPGs after inflammatory stimulation

**Figure 5:**
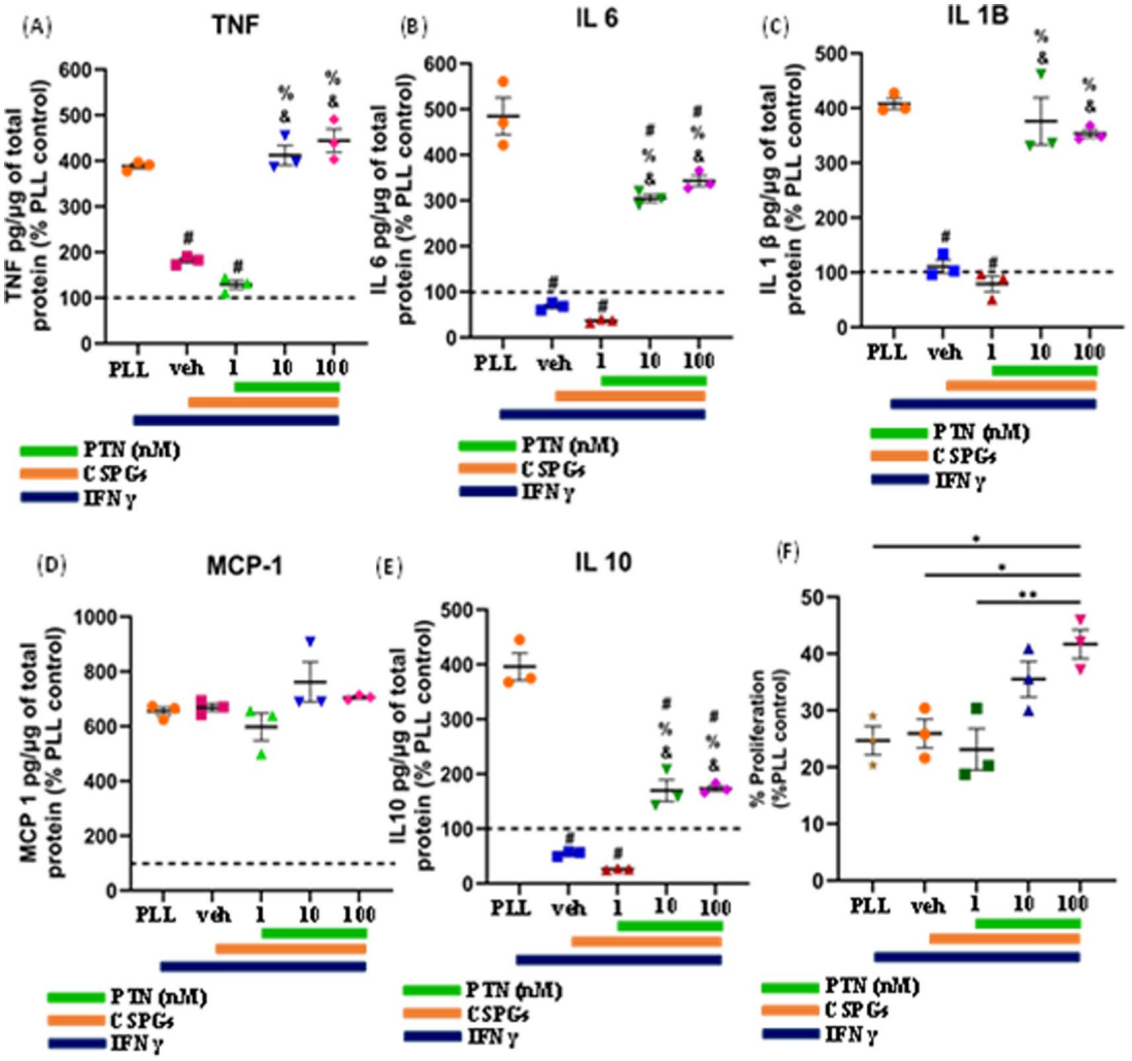
PTN treatment promoted the release of proinflammatory cytokines and the proliferation of IFN-γ-stimulated microglia. Microglia were treated with IFN-γ and different concentrations of PTN for 72 h. PTN treatment increased the release of **(A)** TNF (F_(4, 10)_ = 83.65 *p <* 0.0001), **(B)** IL-6 (F_(4, 10)_ = 95.57 *p <* 0.0001), **(C)** IL-1β (F_(4, 10)_ = 53.29 *p <* 0.0001), **(D)** MCP-1 (F_(4, 10)_ = 2.244 *p =* 0.1367), **(E)** IL-10 (F_(4, 10)_ = 102.5 *p <* 0.0001), **(F)** Proliferation of microglia (F_(4, 10)_ = 7.626 *p* = 0.0044). **(A–F)** The cytokines released from (poly L Lysine) control were considered 100%, error bars represent the standard error of the mean (SEM), symbols: #—compared to PLL + IFNγ, &—compared to the CSPG +1 nM PTN control and %—compared to the CSPG control, #, &, % represents *p <* 0.05, and symbols *, ** represent *p* < 0.05 and 0.01, respectively. All the data are based on 3 independent experiments with a minimum of 3 technical repeats.

To determine the effect of PTN on microglia in the presence of CSPGs under inflammatory conditions, microglia were cultured on CSPGs matrix (5 μg/mL) and treated with different concentrations of PTN (1 nM, 10 nM, or 100 nM PTN) and activated with 100 ng/mL IFNγ for 72 h. The media were collected and processed to detect cytokine levels (TNF, IL-6, IL-1B, MCP and IL-10). These data indicate that in the presence of IFNγ, CSPGs reduce the release of proinflammatory cytokines. However, with PTN treatment, the expression of proinflammatory cytokines increased. A significant main effect of PTN treatment on IFNγ-stimulated microglia in the presence of CSPGs was detected for TNF cytokine (F_(4, 10)_ = 83.65 *p* < 0.0001) (Figure 5A), IL-6 (F_(4,10)_ = 95.57 *p <* 0.0001) (Figure 5B), IL-1β (F_(4,10)_ = 53.29 *p <* 0.0001) (Figure 5C), and IL-10 (F_(4,10)_ = 102.5 *p <* 0.0001) (Figure 5E). For these cytokines, concentrations of 10 nM and 100 nM are typically associated with greater release of inflammatory cytokines. The main effect of PTN on the proliferation of IFN*γ*-stimulated microglia was detected (F_(4, 10)_ = 7.626, *p* = 0.0044; Figure 5G), with the greatest effect occurring at 100 nM PTN.

### 3.5 Expression of MMPs from PTN-treated microglia in the presence of CSPGs

**Figure 6:**
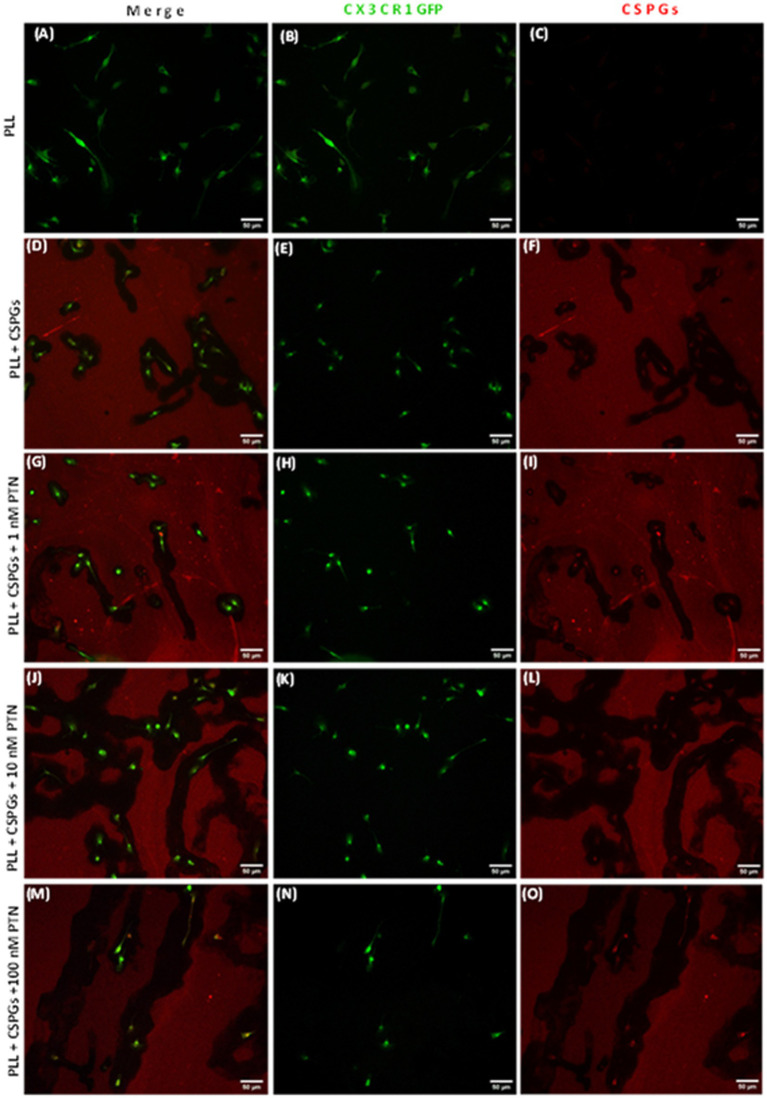
PTN treatment promoted the cleavage of CSPGs by microglia. Microglia from CX3CR1-GFP transgenic mice were cultured on a CSPG matrix, incubated with different concentrations of PTN for 72 h, CSPGs were stained with a CS56 antibody, and the results were detected with Alexa 647 (red). The black area surrounding microglia is indicative of cleaved CSPGs. **(A–C)** Microglia cultured on a polylysine (PLL) matrix. **(D–F)** Microglia cultured on the CSPG matrix. **(G–I)** Microglia were cultured on the CSPG matrix and treated with 1 nM PTN. **(J–L)** Microglia were cultured on a CSPG matrix and treated with 10 nM PTN. **(M–O)** Microglia were cultured on a CSPG matrix and treated with 100 nM PTN.

**Figure 7:**
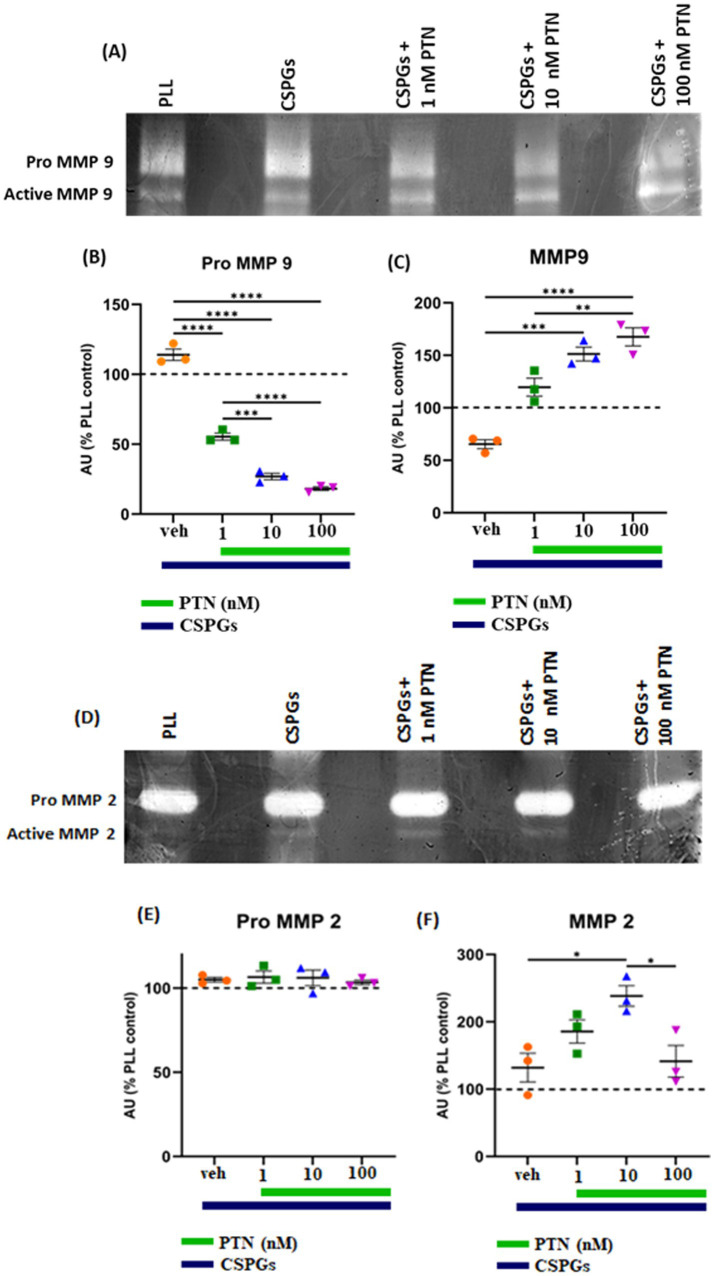
PTN treatment increased the abundance of MMP-9 and MMP-2 in microglia. MMP-9 and MMP-2 activity was detected via gel zymography of conditioned media from microglia treated with or without PTN. The intensities of the white bands for **(A)** pro-MMP 9, active MMP 9, **(D)** pro-MMP 2, and active MMP 2 were calculated via ImageJ software. The intensities of PLL control were considered 100% for pro-MMP9, active-MMP9, pro-MMP2 and active-MMP2. **(B)** PTN significantly reduces the release of pro-MMP 9 from microglia (F_(3, 8)_ = 250.7 *p <* 0.0001). **(C)** PTN significantly increased active MMP 9 levels (F_(3, 8)_ = 38.54 *p <* 0.0001). **(E)** PTN treatment did not affect pro-MMP 2 expression (F_(3, 8)_ = 38.54 *p =* 0.0993). **(F)** PTN treatment significantly increased MMP 2 levels (F_(3, 8)_ = 6.154 *p =* 0.0179). **(B,C,E,F)** Error bars represent the standard error of the mean (SEM), and the symbols *, ** and ***/**** represent *p* < 0.05, 0.01, and 0.001, respectively. All the data are based on 3 independent experiments with a minimum of 3 technical repeats.

**Figure 8:**
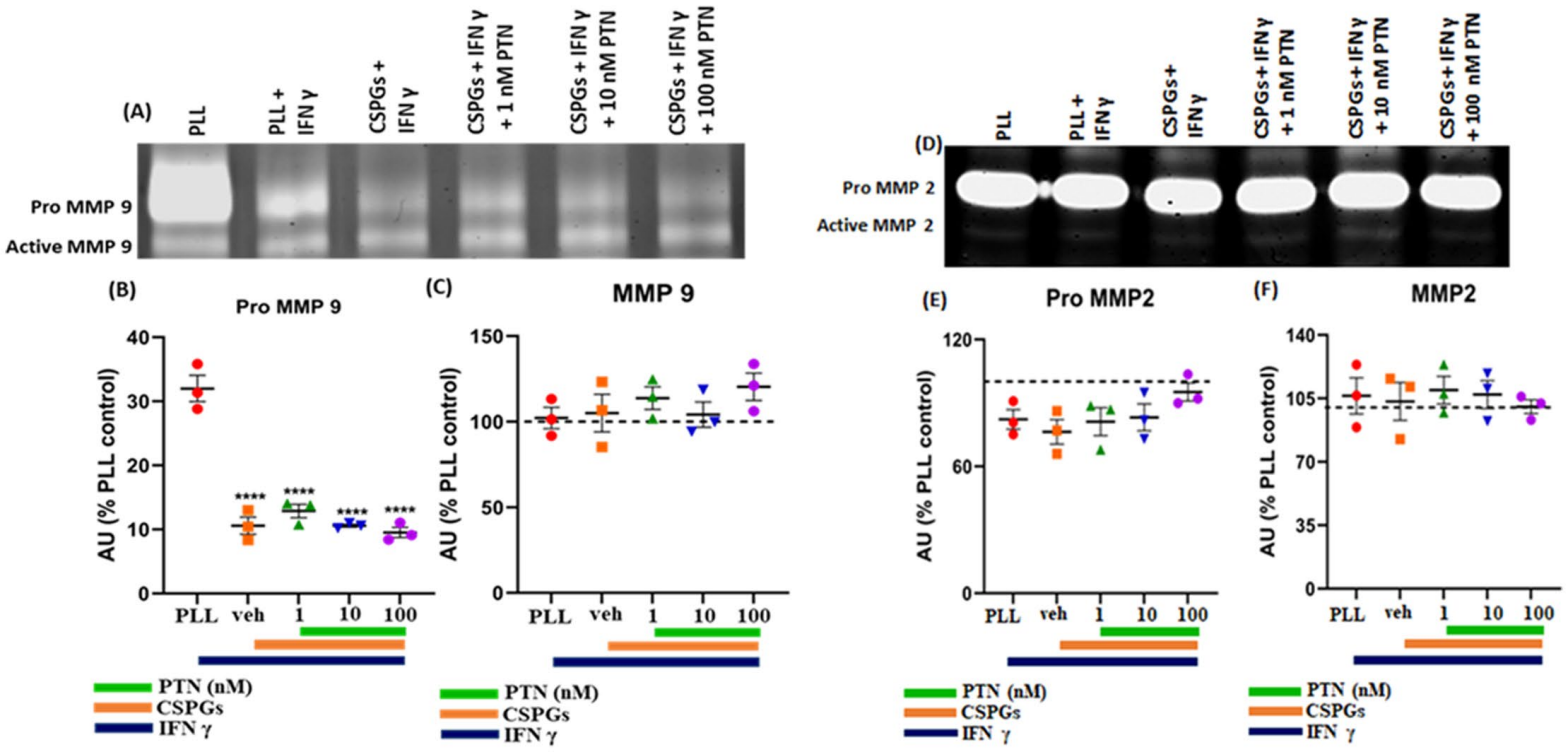
Effects of PTN treatment on the abundance of MMP-9 and MMP-2 in IFN-γ-stimulated microglia. The activities of MMP-9 and MMP-2 were detected via gel zymography of conditioned media from IFN-γ-stimulated microglia treated with or without PTN. The intensities of the white bands for **(A)** pro-MMP 9, active MMP 9, **(D)** pro-MMP 2, and active MMP 2 were calculated via ImageJ software. The intensities of PLL control were considered 100% for pro-MMP9, active-MMP9, pro-MMP2 and active-MMP2. **(B)** PTN significantly reduced the release of pro-MMP-9 from microglia (F_(4, 10)_ = 58.32, *p <* 0.0001). **(C)** PTN did not affect the released active MMP 9 (F_(4, 10)_ = 0.9243 *p =* 0.4873), **(E)** pro-MMP 2 (F_(4, 10)_ = 1.539 *p =* 0.2640) or **(F)** active MMP 2 (F_(4, 10)_ = 0.1837 *p =* 0.9416) levels. **(B,C,E,F)** Error bars represent the standard error of the mean (SEM), and the symbols **** represent *p* < 0.001. All the data are based on 3 independent experiments with a minimum of 3 technical repeats.

MMPs are proteolytic enzymes released from cells that modify the extracellular matrix environment and may reduce the inhibition of growth by CSPGs. Activated microglia and macrophages express MMPs during infection (Rosenberg et al., 2001; Hsu et al., 2006; Rolls et al., 2008) and various CNS disorders, such as multiple sclerosis (MS), cerebral aneurysms, strokes, epilepsy, Alzheimer’s disease (AD), Parkinson’s disease (PD), and brain tumors (Rempe et al., 2016). Here, after 72 h of PTN treatment, media from different treatment concentrations were collected, and the cells were then fixed with 5% formalin and immunostained for CSPGs. CSPG immunostaining (Figure 6) suggested that microglia can degrade CSPGs, leaving dark (unlabeled) paths through the CSPG matrix (Figures 6D–F). With PTN treatment, the unstained area (dark paths) surrounding microglia appeared to increase with 1 nM PTN treatment (Figures 6G–I), followed by 10 nM PTN (Figures 6J–L) and 100 nM PTN (Figures 6M–O). To confirm the involvement of MMPs from microglia in the degradation of CSPGs in the matrix, the conditioned media were concentrated using ampicon and then subjected to SDS–PAGE under non-reducing conditions in gels with 0.1% (w/v) gelatin. The gels were then incubated to determine the proteolytic activity of MMP 9 and MMP 2 by staining with Coomassie Brilliant Blue, with white bands corresponding to the molecular weights of MMP 9 and MMP 2 (Figures 7A,D). There was a significant effect of treatment on the amount of pro-MMP9 released from microglia (F_(3, 8)_ = 250.7 *p <* 0.0001), and *post hoc* tests revealed that the greatest effects occurred at higher concentrations of PTN (10 nM PTN, 100 nM PTN) (Figure 7B). There was a significant effect of treatment on the expression of active MMP 9 (F_(3, 8)_ = 38.54 *p <* 0.0001), and post hoc analysis suggested that there was a significant dose-dependent increase in the expression of MMP 9 with 100 nM PTN (Tukey’s *p* = 0.0070). There was no significant change in the expression of pro-MMP 2 with PTN treatment (Figure 7E), but there was a significant main effect on the expression of active MMP 2 with PTN treatment (F_(3, 8)_ = 6.154 *p* = 0.0179), with peak expression at 10 nM PTN (Figure 7F). Notably, IFNγ is known to inhibit the release of MMP9 (Ma et al., 2001). The effect of PTN on the release of the MMP in IFN-γ-stimulated microglia was investigated. A significant effect of CSPGs treatment on the release of pro-MMP-9 (F_(4, 10)_ = 58.32 *p <* 0.0001) was detected, although post hoc tests on different doses did not reach significance with PTN treatment (Figures 8A,B). PTN treatment had no main effect on active MMP9 levels (Figure 8C). Similarly, the release of pro-MMP-2 and active MMP-2 was not altered by PTN treatment (Figures 8D–F).

## 4 Discussion

### 4.1 PTN overcomes the inhibitory effects of CSPGs on OPC differentiation

CSPGs are among the major components of the extracellular matrix in the CNS (Siebert and Osterhout, 2011). CSPGs are known to inhibit the migration of OPCs, the differentiation of OPCs to mature oligodendrocytes and myelination (Pendleton et al., 2013; Dyck and Karimi-Abdolrezaee, 2014; Lau et al., 2012; Gorter and Baron, 2020). Our data support previous data suggesting that in the presence of CSPGs, OPCs differentiate until they reach premyelinating oligodendrocytes expressing O4 protein (Sun et al., 2017) but cannot further differentiate into mature oligodendrocytes. The inhibition of differentiation into MBP-expressing oligodendrocytes is apparent, with a lack of process outgrowth. As undifferentiated OPCs surrounding the lesion area can be harnessed to improve remyelination, there is a need to identify compounds that can overcome the inhibitory nature of CSPGs and induce their differentiation into mature oligodendrocytes. In this study, we investigated the effect of PTN on the differentiation of OPCs into oligodendrocytes in the presence of inhibitory CSPGs. Previously, PTN was shown to differentiate OPCs into mature oligodendrocytes (Kuboyama et al., 2015). In the present study, we found that OPCs cultured on CSPGs matrix upon treatment with different concentrations of PTN, even the lowest concentration of PTN (1 nM), increased OPC outgrowth. OPCs treated with higher concentrations of 10 nM PTN and 100 nM PTN presented increased expression of O4 and MBP, with complex cellular process outgrowth morphology resembling that of differentiated oligodendrocytes, suggesting that at higher concentrations of PTN, OPCs differentiate into oligodendrocytes even in the presence of CSPGs. The percentage of PDGFR *α* cells in 10 nM PTN-treated conditions was 9.32%, which increased to 45.54% in 100 nM PTN-treated conditions, indicating that the 10 nM PTN concentration favors the differentiation of OPCs (Kuboyama et al., 2015; Tanga et al., 2019) and that the 100 nM PTN concentration favors the proliferation and differentiation of OPCs, potentially by directly binding to and inactivating PTPRZ1 signaling (McClain et al., 2012; Kuboyama et al., 2015; Tanga et al., 2019). Thus, these data suggest that higher concentrations can promote the survival and differentiation of OPCs in the presence of CSPGs. However, one potential contribution to the greater number of PDGFR-positive cells at 100 nM PTN could also be that PTN blocks the binding of CSPGs to their receptors on OPCs (Wang, 2020), allowing more OPCs to remain attached to the matrix, as CSPGs signaling disrupts actin filament polymerization and can inhibit the binding of cells to the matrix (Avram et al., 2014; Jin et al., 2018). Furthermore, PTN has been shown to promote neurite outgrowth by overcoming the inhibition mediated by CSPGs (Gupta et al., 2023; Paveliev et al., 2016), and our data suggest that PTN has the potential to differentiate OPCs into oligodendrocytes; thus, PTN has the potential to remyelinate regenerated neurons. More experiments are needed to verify these findings.

### 4.2 PTN increases the complexity of the cellular outgrowth processes of OPCs and oligodendrocytes

PTN not only increased the number of differentiated cells but also induced process outgrowth from OPCs and oligodendrocytes. Qualitatively, in the absence of PTN, OPCs tend to have a bipolar morphology in CSPG matrices, whereas with PTN treatment, the morphology of OPCs becomes complex, with extensive process outgrowth. For PDGFR*α*-positive cells subjected to PTN treatment, the number of intersections per “Sholl circle” increased within a 7 μm to 50 μm radius from the soma. For O4-positive cells marking immature oligodendrocytes, the number of intersections increased within the 13 μm--113 μm radius from the soma, suggesting enhanced cellular process outgrowth. For MBP-positive cells, PTN treatment increased the number of intersections within the 13 μm to 65 μm radius from the soma. In addition to the alterations in branching patterns, there was an increase in the number of intersections for OPC-, PDGFR*α*-and MBP-positive cells with PTN treatment greater than 1 nM PTN (Figures 2D, 3G). Thus, these findings suggest that higher PTN concentrations are a potential tool for enhancing the differentiation of OPCs and oligodendrocyte growth, making PTN a potential candidate to support the remyelination process in CSPG-rich lesion areas.

### 4.3 PTN reduces the release of proinflammatory cytokines and the MMP in the presence of CSPGs

Inflammation can influence the proliferation and differentiation of OPCs. IFN*γ* plays a pivotal role in shaping the inflammatory microenvironment in AD, PD, MS and stroke (Planas, 2024; Ottum et al., 2015; Monteiro et al., 2017; Tan et al., 2022). The release of IFNγ from activated microglia (Corbin-Stein et al., 2024) induces apoptosis, delays remyelination, and inhibits OPC differentiation and remyelination (Lin et al., 2006; Harrington et al., 2020). Thus, modulating the inflammatory response of microglia may alter the differentiation process, affecting remyelination. Previous studies have shown that PTN and CSPGs can affect the inflammatory activity of microglia. PTN potentiates a more proinflammatory phenotype in response to LPS treatment (Fernández-Calle et al., 2017), whereas in the presence of CSPGs, microglia reduce the expression of cytotoxic TNF-α and NO after LPS treatment (Rolls et al., 2008). Here, we examined the combined effect of PTN on microglial activity in the presence of CSPGs. Notably, CSPGs reduced the expression of proinflammatory cytokines (TNF, IL-6, IL-1β and MCP-1) in non-stimulated microglia. These nonactivated microglia treated with PTN in the presence of CSPGs further reduced the expression of proinflammatory cytokines (TNF, IL-6, IL-1β and MCP-1), especially at relatively high concentrations of PTN (10 nM and 100 nM). These findings suggest that PTN may be a potential agent to reduce the release of proinflammatory cytokines during the chronic phase of injury when microglia are less activated than during acute injury. This reduction in inflammatory signaling in the chronic phase could support remyelination.

In addition to reducing the expression of proinflammatory cytokines, PTN treatment also induced the release of MMP-9 and MMP-2 from nonactivated microglia. MMPs can degrade CSPGs in the glial scar region or adjacent regions. Given that CSPGs are expressed and can inhibit the neuronal growth and differentiation of OPCs into oligodendrocytes (Siebert et al., 2014; Sun et al., 2017; Lau et al., 2012), the ability of PTN to upregulate MMPs may induce a growth-permissive environment. Thus, during chronic injury, when microglia are less activated, PTN may potentiate remyelination and recovery via direct influences on OPCs and oligodendrocytes and by modulating the release of inflammatory cytokines and MMPs by microglia.

### 4.4 PTN enhances the release of inflammatory factors from activated microglia in the presence of CSPGs

During the acute phase of injury, inflammation is mediated by microglia and macrophages (Rolls et al., 2008; Yenari et al., 2010; Chew et al., 2006). Inflammatory signaling is essential during the acute phase of stroke to recruit microglia and macrophages to the damaged area and restrict the spread of infarction to other parts of the brain by forming a glial scar (Anrather and Iadecola, 2016). Here, we modeled early inflammatory conditions in an injury model by stimulating microglia with IFN*γ* (Seifert et al., 2014; Yilmaz et al., 2006). Previous studies have shown that CSPGs reduce the release of TNF-*α* and NO after LPS stimulation (Fernández-Calle et al., 2017), and our data further confirm that CSPGs also reduce the release of proinflammatory cytokines from microglia under IFNγ stimulation. IFN-γ-stimulated microglia, upon treatment with higher concentrations of PTN (10 nM and 100 nM), promoted the release of the pro-inflammatory cytokines PTN (TNF, IL-6 and IL-1*β*) and IL-10 but did not alter the levels of MCP-1. These findings suggest that PTN can increase the inflammatory activity of activated microglia in the presence of CSPGs. PTN may therefore be useful during the acute phase of nervous system injury, as restoring the inflammatory activity of microglia to clear debris and restricts the spread of infarction. As mentioned previously, PTN treatment in the presence of CSPGs can induce the release of MMP 9 and MMP 2, but after IFNγ treatment, PTN does not increase the release of MMP 9 or MMP 2. The lack of released MMP 9 and MMP 2 could be beneficial during the acute stage of stroke, as CSPGs play an important role in forming the glial scar, which acts as a physical barrier for the expansion of the infarct. Thus, PTN may help during the early stage of injury by increasing the release of proinflammatory cytokines and modulating the activity of microglia and astrocytes to form a glial scar. These effects during acute stroke differ from PTN actions during later phases of injury, where reduced release of proinflammatory cytokines and increased release of MMP-9 and MMP-2 can create an environment favorable for neuronal regeneration and OPC differentiation to oligodendrocytes and remyelination.

Thus, the findings of this study suggest that microglia, the primary immune cells of the central nervous system, are pivotal in regulating the inflammatory response during injury or disease. Activation of microglia can occur through the release of IFN-γ, which subsequently triggers the production and release of pro-inflammatory cytokines, including IL-6, TNF-*α*, MCP-1, and IL-1β. In the presence of CSPGs, an inhibitory component of the extracellular matrix, the pro-inflammatory activation of microglia is attenuated, resulting in a less pronounced inflammatory response.

Treatment with PTN, a heparin-binding growth factor, counteracts the CSPG-mediated inhibition and restores the pro-inflammatory activity of microglia under inflammatory conditions. Interestingly, in the absence of a pro-inflammatory stimulus, PTN exhibits anti-inflammatory effects by suppressing the expression of pro-inflammatory cytokines. Additionally, PTN induces the upregulation of matrix metalloproteinases (MMPs), specifically MMP-9 and MMP-2, which play critical roles in extracellular matrix remodeling.

**Figure 9:**
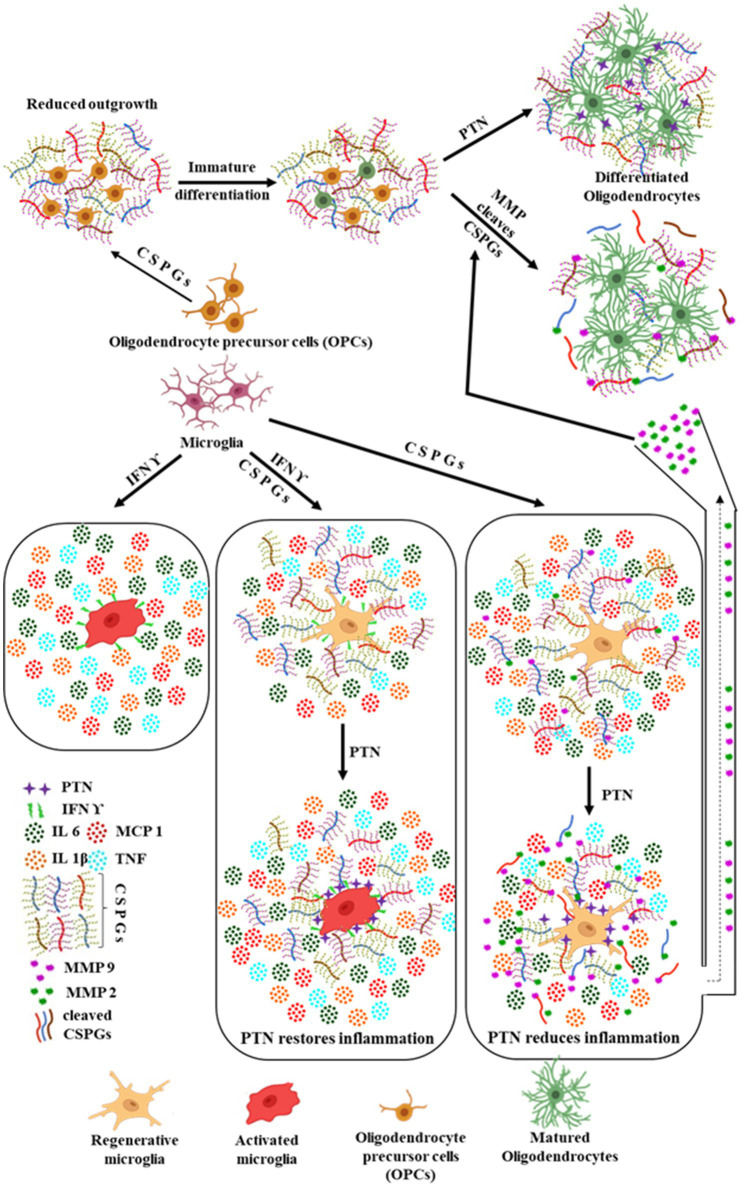
Summarized effect of PTN on OPCs and microglia in the presence of CSPGs. Microglia being the immune cells in the brain can be activated by the release of IFN γ due to any injury in the brain, thus promoting the release of pro inflammatory cytokines. In the presence of CSPGs, this pro inflammatory activated state of microglia is reduced by reduced release of pro inflammatory cytokines and upon treatment with PTN the pro inflammatory activity is restored. In contrast, in the absence of pro inflammatory stimulus, PTN reduced the expression of pro inflammatory cytokines and induced the expression of MMP 9 and MMP 2. PTN also promoted the differentiation of OPCs into oligodendrocytes even in the presence of CSPGs by directly binding to OPCs. Another possible mechanism by which PTN enhances OPC differentiation in the presence of microglia and CSPGs could be through the increased release of MMP-2 and MMP-9. These MMPs can cleave CSPGs, thereby eliminating their inhibitory effect on OPC differentiation. The MMPs released from microglia upon PTN treatment may further degrade CSPGs, facilitating the differentiation process. Figure created using BioRender.com.

Furthermore, PTN facilitates the differentiation of oligodendrocyte precursor cells (OPCs) into mature oligodendrocytes. This process is supported by the activity of MMPs, which cleave CSPGs, thereby mitigating their inhibitory effects on OPC maturation. Collectively, these findings highlight the dual regulatory role of PTN in modulating microglial activity and promoting oligodendrocyte differentiation by altering the CSPG-rich extracellular environment, Figure 9 illustrate the distinct roles of PTN in modulating the activity of microglia in presence and absence of inflammatory milieu (IFN γ), OPCs, and mature oligodendrocytes in these processes, emphasizing the interplay between PTN, CSPGs, and microglial activity in the inflammatory milieu and differentiation cascade.

## 5 Limitations and future directions

Our study quantified specific cytokines involved in inflammation, but this analysis was limited to a subset of secreted factors, and some key signaling molecules that may influence myelination were not assessed. For example, IL 4, IL 13, Transforming growth factor *β* (TGF-β) and leukemia inhibitory factor (LIF) promote oligodendrocyte differentiation and remyelination (Ishibashi et al., 2006; Zhang et al., 2024; Zhang et al., 2019), but were not included in this assessment. On the contrary, IL 6 can have dual effects on OPC differentiation, with lower levels promoting differentiation and higher concentrations inhibiting myelination (Zhang et al., 2024). Expanding the cytokine analysis, and including IL 4, IL 13, TGF-β, and LIF, would provide a more comprehensive understanding of PTN’s effects on microglia in the presence of CSPGs. Additionally, classifying cytokines as strictly pro-inflammatory is an oversimplification, as many cytokines have dual roles depending on their concentration and cellular interactions. TNF-α, commonly associated with inflammation, can also promote remyelination by enhancing oligodendrocyte precursor cell (OPC) proliferation (Ishibashi et al., 2006), while IL 10, traditionally considered anti-inflammatory, modulates microglial activation and supports tissue repair (Zhang et al., 2024). IL 4, typically linked to anti-inflammatory responses, also contributes to remyelination process by promoting differentiation (Zhang et al., 2019). These examples emphasize the need for a future study to explore the effect of these cytokines on microglial activation and myelination in the presence of CSPGs. Moreover, while this study provides significant insights on the potential mechanisms by which PTN modulates microglial activity, promotes oligodendrocyte differentiation, and interacts with CSPGs in a controlled cell culture environment, the relevance of these mechanisms in the context of an intact nervous system remains to be explored. All the data generated in this study is *in vitro* studies, and to facilitate translation, future studies will need to investigate these findings in more complex *in vivo* models of nervous system injury to fully understand the impact of these interventions within an integrated nervous system. Several *in vivo* studies have highlighted its potential therapeutic benefits in other neurological contexts. For instance, PTN has been shown to promote neuroprotection and functional recovery in Parkinson’s disease models by enhancing dopaminergic neuron survival and modulating neuroinflammation (Chertoff et al., 2011; Herradon and Ezquerra, 2009). Additionally, studies with spinal cord injury model suggest that PTN may facilitate axonal regeneration and improved functional recovery (Alsmadi et al., 2018). Given these findings, further exploration of PTN’s role in myelination within these disease or injury models could provide valuable insights into its neuroprotective mechanisms system.

PTN signals through a number of receptors, including protein tyrosine phosphatase β/*ζ* (RPTPζ/PTPRZ), syndecans, nucleolin, neuropilin-1, integrins αVβ3 and αMβ2, N-syndecan receptor, glypican 2, neuroglycan-C, and anaplastic lymphoma kinase (ALK) (Gupta et al., 2023; Paveliev et al., 2016; Wang, 2020). Which of these receptors are most important to its effects on myelination remains to be confirmed. While the literature suggests that PTN signals through PTPRZ in OPCs (Tanga et al., 2019) and RPTPβ/ζ via ERK1/2 pathway in microglia (Fernández-Calle et al., 2020; Miao et al., 2012), identifying other PTN receptors involved in OPC and microglia signaling in the presence of CSPGs will bridge the gap between these foundational findings and their potential therapeutic applications in the treatment of nervous system injuries or diseases.

## Data Availability

The original contributions presented in the study are included in the article/supplementary material, further inquiries can be directed to the corresponding author.
